# Pediatric Cervicofacial Necrotizing Fasciitis: A Case Report Highlighting the Diagnostic Challenges and Management in a Child Without Preceding Trauma or Infection

**DOI:** 10.1002/ccr3.71294

**Published:** 2025-11-26

**Authors:** Anza Muhammad Mohsin, Zahra Anas, Zarlish Khan, Muhammad Abdullah Bin Fahad

**Affiliations:** ^1^ Department of Internal Medicine Dr Ruth KM Pfau Civil Hospital Karachi Pakistan; ^2^ Department of Internal Medicine DOW University of Health Sciences Karachi Pakistan; ^3^ Shaheed Syed Nazrul Islam Medical College Hospital Dhaka Bangladesh; ^4^ Voice of Doctors Research School Dhaka Bangladesh

**Keywords:** epilepsy, necrotizing fasciitis, non‐transfusion‐dependent thalassemia, pediatric

## Abstract

Necrotizing fasciitis (NF) is a rare but serious life‐threatening condition in children that can be easily overlooked due to its atypical presentation and absence of common risk factors. This case involves a 10‐year‐old girl with a history of non‐transfusion‐dependent thalassemia and epilepsy who developed cervicofacial NF without any prior trauma or infection. The case highlights the importance of early recognition and prompt surgical intervention. In patients with underlying conditions, the rapid progression of NF underscores the need for a high index of suspicion when faced with unusual facial swelling or skin changes. Early surgical debridement and appropriate antibiotics are critical to improving outcomes and minimizing complications in pediatric NF cases.

## 1 Introduction

Necrotizing Fasciitis is a life‐threatening condition that accounts for 0.03% of hospitalizations in children across the globe. This condition involves polymicrobial infection and necrosis of the subcutaneous and fascial planes that require urgent debridement. It is rare in this demographic compared to adults because of the smaller number of comorbidities, including immunosuppressive disorders like type 2 Diabetes Mellitus, and the rarity of NF in children leads to misdiagnosis and difficulty in appropriate management and investigations [1, 2].

Necrotizing fasciitis of the cervical and fascial planes is uncommon and mostly involves the lower limbs and chest walls; however, when it involves the cervical plane, it spreads to the face and mediastinum, particularly in children, unlike adults, with a higher prevalence of 15%–20% [1, 3]. There are multiple inciting factors; however, in the pediatric population, it is either odontogenic or varicella [3]. Polymicrobial infections with aerobic and anaerobic microorganisms are the most prevalent in cervicofacial necrotizing fasciitis, but other organisms include Streptococcus, Staphylococcus, Clostridium, and fungi, such as candida [3].

This case report presents a rare case of a child with a plethora of coexisting conditions such as non‐transfusion‐dependent thalassemia, epilepsy, and localized facial swelling that progressed to the neck along with the appearance of blisters without any preceding factors of trauma, varicella rash, laceration, or dental procedure. This case was diagnosed with necrotizing fasciitis after biopsy and histopathological examination. Our case highlights this phenomenon in the pediatric population, which is increasingly misdiagnosed as cellulitis that results in a delay of medical and surgical intervention, increasing mortality and morbidity.

## 2 Case History/ Examination

**FIGURE 1:**
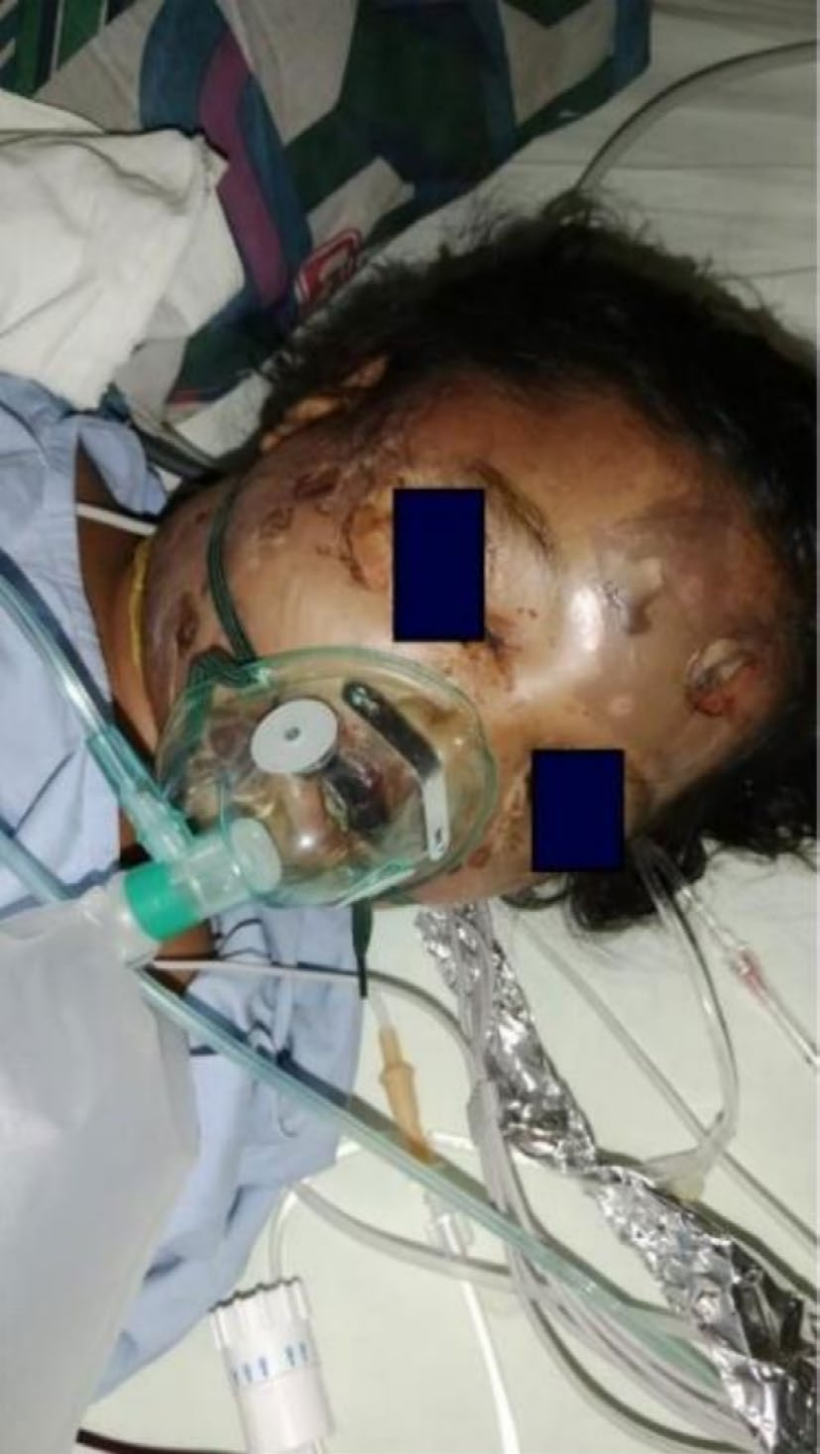
Cervicofacial necrotizing fasciitis at the time of admission before debridement.

A 10‐year‐old girl with a known history of non‐transfusion‐dependent thalassemia since the age of 1 year, which is positive for IVS1‐5 IVSI‐5 mutation. She was brought to the Children's Emergency Department with localized facial swelling, blisters, and difficulty in opening the eye in the affected area. The swelling appeared suddenly, initially affecting the area below her right eye, but quickly spread to the entire face and neck. Skin changes accompanied the swelling, including dark patches around the lips, as shown in Figure 1. Within 24 h, blisters developed over her forehead and cheeks, and she lost consciousness. There was no history of fever, discharge, trauma, dental procedures, or snake or scorpion bite.

Her medical history included a diagnosis of thalassemia major at the age of one, for which she was treated with hydroxyurea, eliminating the need for blood transfusions. At age five, she was diagnosed with epilepsy following multiple seizures, and a CT scan revealed ex vacuo dilatation of the ventricles and generalized cerebral atrophy (Figures 3 and 4). She was prescribed levetiracetam and topiramate for epilepsy management. Despite her condition, she achieved all developmental milestones.

Upon admission to the pediatric ICU, the patient was hemodynamically stable but with a massive swelling in the facial and cervical region, measuring 22 cm in diameter, along with blisters that had begun to rupture, releasing a bloody discharge. The swelling was firm, non‐fluctuating, and was associated with hyperemic skin changes. Necrotic patches were also observed on the upper lip and the right angle of her mouth.

Neurological examination revealed a Glasgow Coma Scale (GCS) score of 12/15 with no eye opening due to extensive swelling and pressure in response to the swelling. However, her motor and verbal responses were intact. Her sensory system, muscle tone, strength, and reflexes were normal, with bilaterally reactive pupils. Abdominal examination revealed non‐tender hepatomegaly (10.5 cm) and splenomegaly (1 cm below the costal margins). Cardiovascular and respiratory examinations were unremarkable.

## 3 Methods

On admission, the patient's vital signs were as follows: temperature 98.6°F; blood pressure, 109/60 mmHg; pulse, 120 beats per minute; respiratory rate, 25 breaths per minute; capillary refill time, < 2 s; and oxygen saturation, 98% on a non‐rebreather mask with 10 L of oxygen because of laborious breathing and decreasing saturation with hoarseness and respiratory distress. Laboratory tests revealed anemia (hemoglobin 6 g/dL), a total leukocyte count of 5.1 × 10^9^/L (83% neutrophils, 12% lymphocytes), and a platelet count of 655 × 10^9^/L. Liver function tests showed elevated bilirubin levels (total bilirubin 7.6 mg/dL, direct 5.1 mg/dL, indirect 2.5 mg/dL), secondary to thalassemia, while aspartate transaminase (AST) and alkaline phosphatase levels were within normal ranges. Urinalysis revealed microscopic hematuria with 8–10 red blood cells per high‐power field and 1–2 leukocytes per high‐power field. Blood cultures showed Klebsiella growth, but pus, eye swabs, and urine cultures were negative as shown in Table 1.

**TABLE 1:** Various lab parameters on admission.

Hematology	Day 1	Day 2	Day 3	Normal range	Units
Hb	6	11.2	10.2	11.5–15.5	g/dL
MCV	69.5	71.7	73.3	80.0–100.0	fL
HCT	19.8	31.9	31		
TLC	5.1	13.4	6.1	4.0–10.0	×10^9^/L
Lymphocytes	12	7	11	20.0–40.0	%
Neutrophils	83	91.5	88	40.0–80.0	%
Platelets	655	426	269	150.0–450.0	×10^9^/L
CRP	168	66.9	14.3	< 0.3	mg/dL
**Urine D/R**	Ph:6, specific gravity: 1.01, protein trace, normal qaunity of urinobilogen, trace of blood, red cells: 8–10/hpf and pus cells 1–2/hpf	—	—		
**Basic metabolic profile**					
**Component**				**Normal range**	**Units**
BUN	33	24	32	7–20	mg/dL
Cr	0.5	0.5	0.6	0.7–1.3	mg/dL
Sodium	144	153	148	134–144	mEq/L
Potassium	4.6	4.1	4.4	3.5–5.2	mEq/L
Chloride	112	121	109	96–105	mEq/L
Calcium	8.1	8.4	9.2	8.7–10.2	mg/dL
Magnesium	3.0	2.0	1.8	1.7–2.2	mg/dL
Phosphate	4.8	2.9	4.1	2.5–4.5	mg/dL
**Liver function tests**					
**Component**				**Normal range**	**Units**
ALT	29	30	30	7–55	U/L
ALP	62	76	77	40–129	U/L
Total bilirubin	7.6	2.8	1.6	0.1–1.2	mg/dL
Direct bilirubin	5.1	1.4	—		
Indirect bilirubin	2.5	1.4	—		

The patient was initially suspected of having an insect bite, such as a scorpion bite, due to localized swelling. However, further evaluation led to a diagnosis of necrotizing fasciitis of the cervicofacial region with oral involvement, with disease process showing intermediate Stage 2 in the form of blisters and Late stage 3 of necrotizing fasciitis in the form of necrotic patches.

**FIGURE 2:**
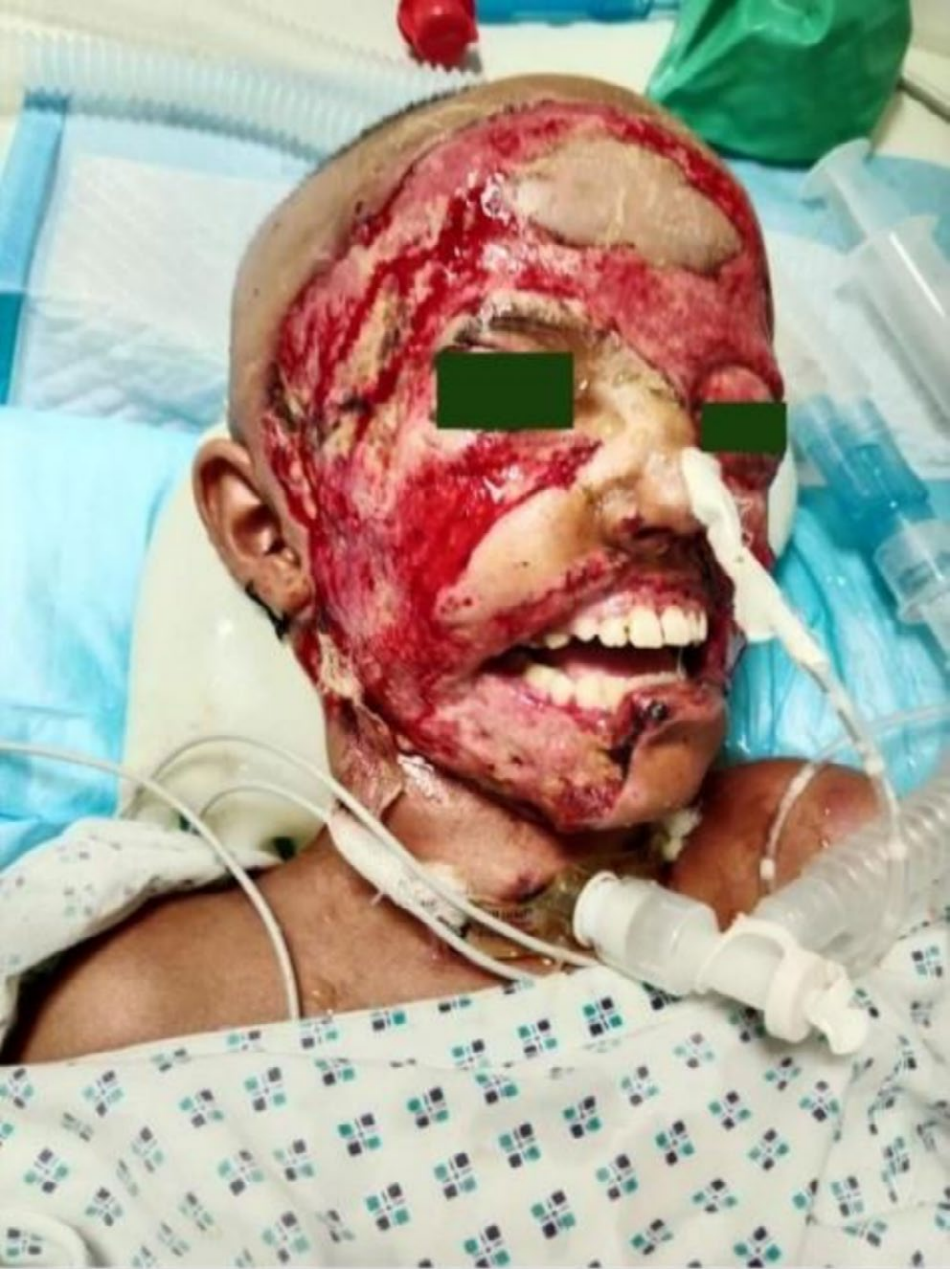
Cervicofacial necrotizing fasciitis post debridement along with tracheostomy.

**FIGURE 3:**
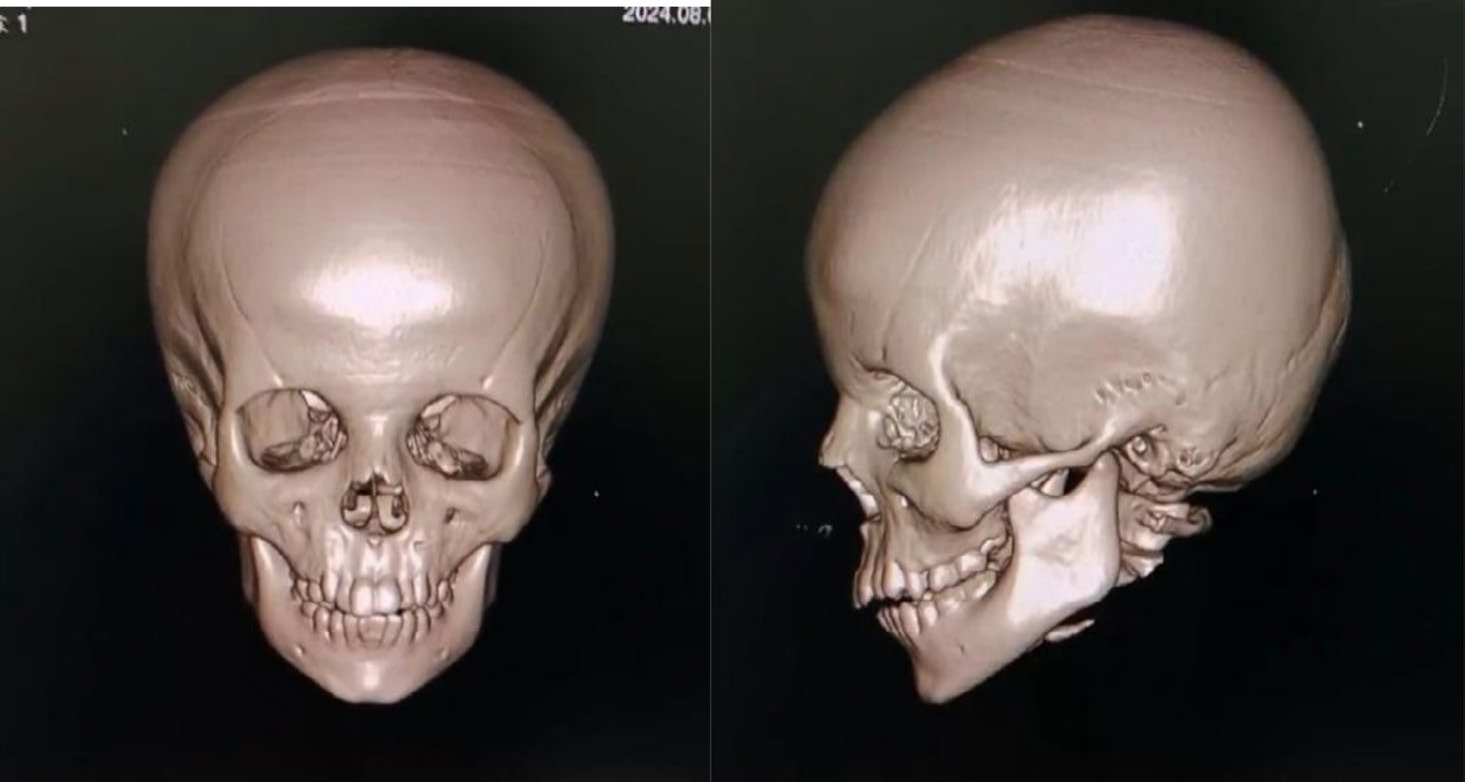
3D CT scan showing only soft tissue edema without any bone involvement.

**FIGURE 4:**
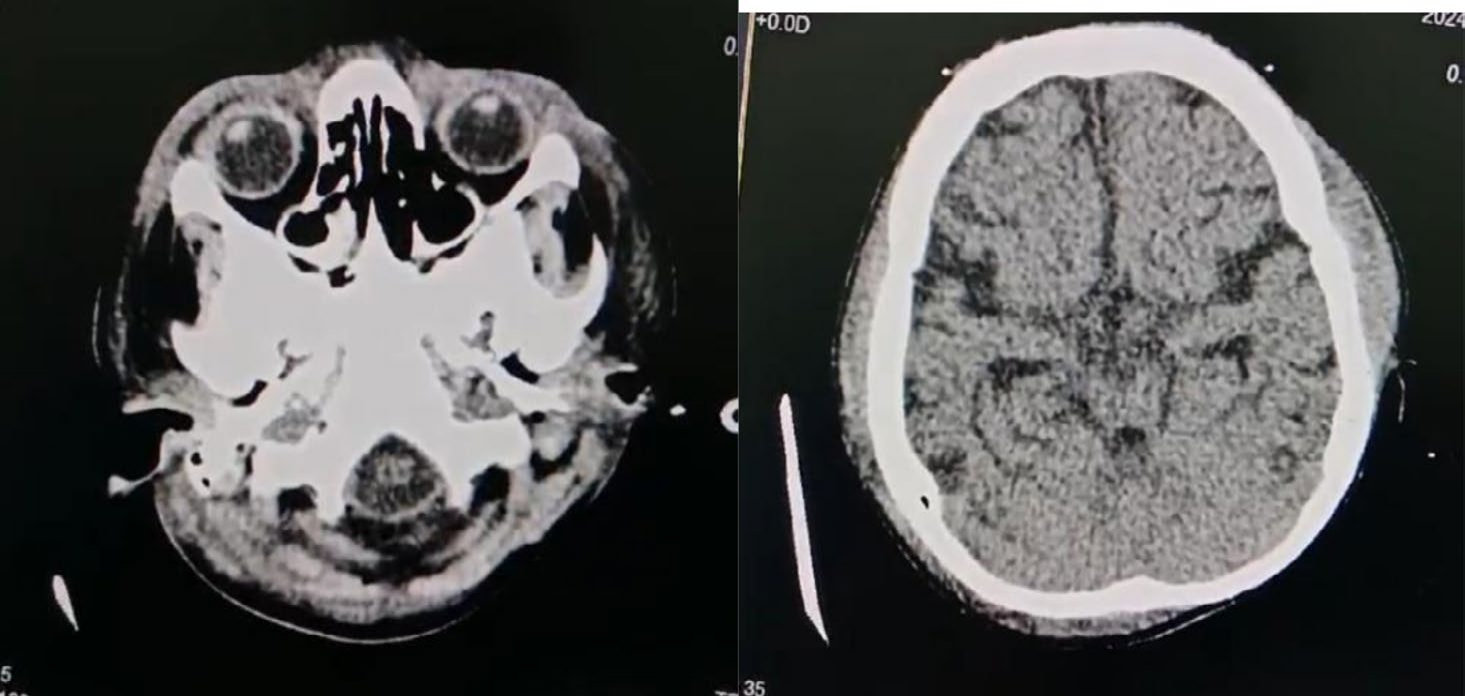
Ct scan brain showing soft tissue thickening with few air loculi observed along the plane of the right premaxillary and zygomatic regions.

Given the facial swelling and necrotic patches around the lips and mouth, an oral maxillofacial team was consulted. A 3D CT scan based on their recommendation revealed soft tissue oedema without bone involvement, confirming necrotizing fasciitis shown in Figures 2 and 3. CT scan of the brain was also done without contrast, showing soft tissue thickening with few air loculi observed along the plane of the right premaxillary and zygomatic regions and inappropriate cerebral atrophy as an incidental finding, as shown in Figure 4. Urgent debridement and biopsy were then performed. Ophthalmology cleared the patient for eye involvement; therefore, debridement was limited to the affected areas, sparing the eye. Histopathology revealed ulceration of the skin, dermis, and subcutaneous tissue with dense mixed inflammatory granulation tissue and necrotic debris, confirming the diagnosis of necrotizing fasciitis. Tissue cultures revealed the presence of *Candida tropicalis* and Aspergillus flavus; however, no other microorganisms were detected. Moreover, rapid biopsy could not be carried out as our tertiary care hospital does not have these resources.

As the infection spread to the cervical region, secondary to laryngeal edema due to which tracheostomy was carried out during her first debridement to secure the airway. LRINEC score was found to be 6. Swelling extended up to the upper part of the neck in the anterior triangle of the neck, although no necrotic tissues were observed there. A 2.5 cm incision was made above the suprasternal notch with fascia and strap muscles separated, trachea identified, and an incision was given with a 5.0 fr tracheostomy tube inserted and hemostasis secured. After the procedure, dressing was done with sufra tulle (antibacterial dressing) once daily as recommended by the OMF department.

Based on the culture and biopsy results, the patient was provided with a regimen of intravenous antibiotics, including meropenem (1450 mg every 8 h), vancomycin (720 mg every 8 h), colistimethate sodium (100 mg loading dose followed by 35 mg), and amphotericin (1 mg/kg once daily). Dexamethasone (5.5 mg every 6 h) was administered for hoarseness and to decrease laryngeal oedema. The patient was also placed on epinephrine support at 0.1 mg/kg/h over 24 h as the patient went into septic shock. Fluid maintenance included 1600 mL of 0.9% dextrose saline administered at 66 mL/h. Topical polymyxin was administered every 8 h, and intravenous nalbuphine (3 mg) was administered for pain relief.

After completing the course of antibiotics, a plastic surgery team was consulted, and the patient underwent reconstructive surgery with grafting.

## 4 Discussion

Necrotizing fasciitis is a life‐threatening soft tissue infection characterized by widespread destruction of the fascia and subcutaneous tissue, with rapid progression of the disease. Because NF is uncommon and lacks early pathognomonic indications, it is one of the most difficult infections for doctors to diagnose and treat promptly. NF also affects children despite being more prevalent in adults. Although necrotizing fasciitis has been the focus of significant research in adults, our understanding of the condition in children remains limited. In previously healthy children, pediatric NF typically manifests as oedema, induration, erythema, or sepsis. Diagnosing is challenging because clinical symptoms may range from a major presentation to subtle and sneaky progression [4, 5]. This type of infection is more common in individuals with weakened immune systems, including those with conditions like diabetes, cancer, alcoholism, vascular disorders, organ transplants, HIV, or neutropenia. The disease most commonly affects the trunk, extremities, and perineum, while involvement of the head and neck region is rare, accounting for only 1%–10% of cases. Most cases of cervicofacial necrotizing fasciitis originate from odontogenic sources. When the disease first appears, it spreads quickly over adjacent facial areas, leading to characteristic necrosis of the fascia and skin. Considerable damage may also result from bone, muscle, and gland tissue necrosis. Although it can be challenging to identify these infections in their initial stages, they spread quickly and require intensive treatment to reduce the high morbidity and fatality rates associated with them [6, 7].

Pfeifle et al. reported two cases. In the first case, a 5‐year‐old Caucasian boy developed a varicella infection. Six days later, a severe secondary infection appeared at the site of a back lesion and was initially treated with antibiotic ointment. As the infection worsened, he was taken to the emergency department, where sepsis was suspected. Upon examination, the lesion had increased, and palpation revealed palpable lymph nodes in the right axilla. After suspecting NF and confirming the diagnosis with an ultrasound, debridement was performed. In our case, the child had no recent history, signs, or symptoms of exanthems.

The second case was a 4‐year‐old boy who presented to the emergency department with a high‐grade complaint. In addition to having a high fever (40°C), he had growing redness and oedema in his right lower leg when he arrived at the emergency room. He recalled being bitten a few days before by an insect, most likely a mosquito. Initially, cellulitis was suspected; however, as the patient's clinical condition deteriorated, a final diagnosis of necrotizing fasciitis was made after debridement. In contrast to our case, the child had no history of insect bites [8].

King et al. reported a case in which a child, after falling off his bike, a 5‐year‐old youngster who had been in good health, bit his right lower lip and skinned his chin. After 24 h, he experienced fever, emesis, nausea, minor chin and lower lip swelling, and abdominal pain. He was initially admitted to the pediatric ICU without airway distress. Initial evaluation revealed marked tense oedema of the right face, cheek, and neck, with poorly defined erythema, severe neck tenderness, and cheek anesthesia. Necrotizing fasciitis was suspected, and debridement and tracheostomy were performed [9]. In our case, the child did not have a history of trauma. Lodhia et al. presented a case of a 5‐week‐old female infant with a 4‐day history of fever (40°C) and a rapidly progressing ulcer on the right lower jaw. The lesion began as a skin darkening, progressing to reddish discolouration, ulceration, and serosanguinous discharge, accompanied by hard, tender, warm swelling extending to the cheek, preauricular, and neck regions. She was born vaginally (3 kg) to an HIV‐positive mother on long‐term ART (tenofovir, lamivudine, and efavirenz) and had been receiving prophylactic nevirapine since birth. Suspected necrotizing fasciitis of the jaw prompted surgical debridement under general anesthesia [10]. In our case, the mother did not have HIV infection or other illnesses.

## 5 Conclusion

This case highlights the challenges in diagnosing and managing necrotizing fasciitis (NF) in pediatric patients, particularly when the presentation lacks typical risk factors or preceding infections. Rapid progression of the disease necessitates a high level of suspicion and prompt intervention to reduce the risk of severe morbidity and mortality. Our case contributes to the growing body of knowledge on NF in children, emphasizing the importance of early recognition, timely surgical intervention, and supportive care in improving outcomes. Given the rarity of NF in the head and neck region, this case underscores the need for clinicians to remain vigilant and consider NF in their differential diagnosis, even when there is no history of trauma or infection. Early intervention remains the key to improving survival and reducing complications.

## Author Contributions

**Anza Muhammad Mohsin:** conceptualization, data curation, formal analysis, investigation, methodology, writing – original draft. **Zahra Anas:** conceptualization, data curation, formal analysis, methodology, resources, writing – original draft. **Zarlish Khan:** data curation, formal analysis, investigation, methodology, visualization, writing – original draft. **Muhammad Abdullah Bin Fahad:** formal analysis, resources, supervision, visualization, writing – review and editing.

## Disclosure

Provenance and peer review: Not commissioned, externally peer reviewed.

## Ethics Statement

The authors have nothing to report.

## Consent

Written informed consent was obtained from the patient to publish this report in accordance with the journal's patient consent policy.

## Conflicts of Interest

The authors declare no conflicts of interest.

## Data Availability

The authors have nothing to report.
